# Supplementary material for: Spatial Attention Modulates Neuronal Interactions between Simple and Complex Cells in V1
Source: Int J Mol Sci. 2023 May 4;24(9):8229. doi: 10.3390/ijms24098229 (PMC10179430; doi:10.3390/ijms24098229)
Supplement: Supplementary file 1 [file ijms-24-08229-s001.zip › ijms-2345848-supplementary.pdf]

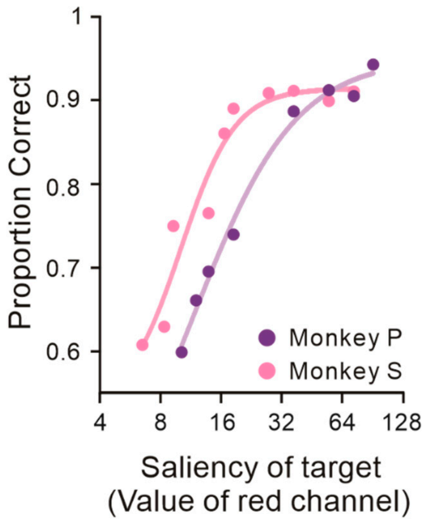

Supplementary Figure S1. Performance curves of two macaque monkeys across sessions. Individual points represent the averaged proportion correct at each redness value (monkey P: purple; monkey S: pink). Solid lines represent logistic function fits to the data.

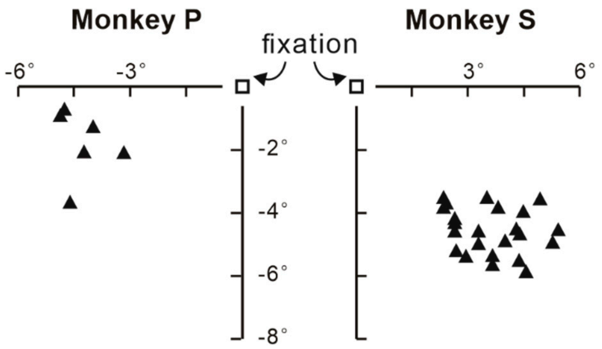

Supplementary Figure S2. The position of recorded neurons' RF centers (monkey P: left; monkey S: right) and the fixation point (hollow square).

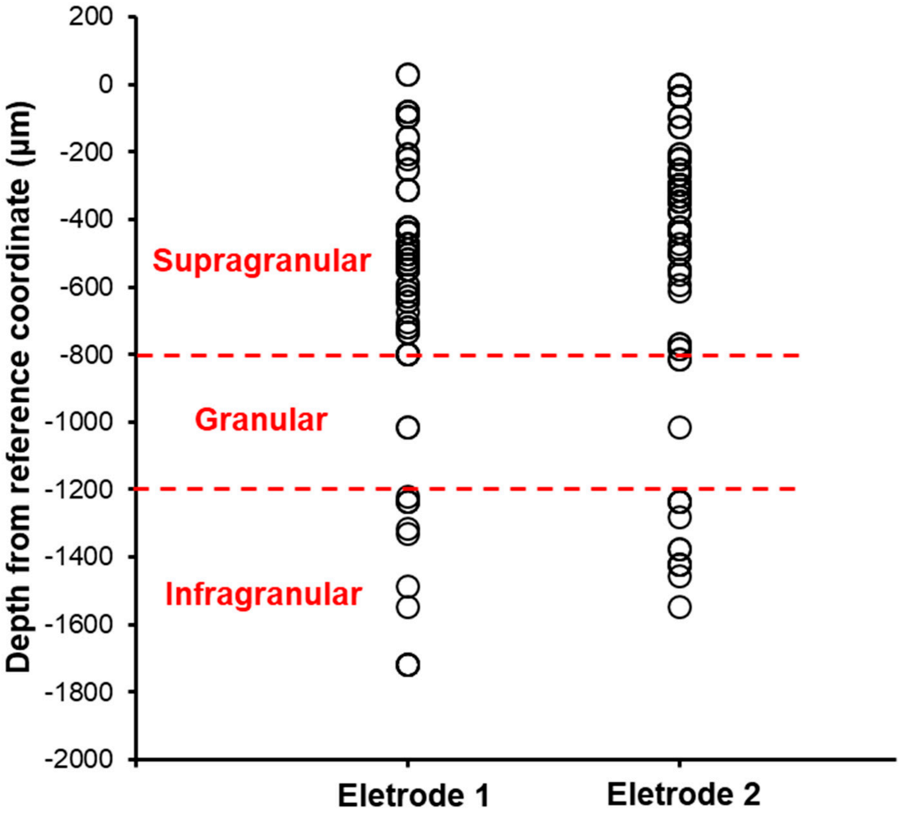

Supplementary Figure S3. Recorded neurons' depth from reference coordinate recorded by two electrodes.
